# Differential effects of olive oil, soybean oil, corn oil and lard oil on carbon tetrachloride-induced liver fibrosis in mice

**DOI:** 10.1042/BSR20191913

**Published:** 2019-10-11

**Authors:** Yanan Gao, Xuguang Li, Qiang Gao, Li Fan, Haobin Jin, Yueping Guo

**Affiliations:** 1 Key Laboratory of Tropical Translational Medicine of Ministry of Education; Hainan Key Laboratory for Research and Development of Tropical Herbs; School of Pharmacy, Hainan Medical University, Haikou, Hainan 571199, P.R. China; 2 Department of Pharmacology, Harbin Medical University, Harbin 150081, China; 3 Department of Anesthesiology, The First Affiliated Hospital of Hainan Medical University, Haikou, Hainan 570102, P.R. China

**Keywords:** Corn oil, Lard oil, liver fibrosis, Soybean oil

## Abstract

Olive oil could attenuate carbon tetrachloride (CCl_4_) induced liver fibrosis (LF) in mouse model. The present study aimed to evaluate the effects of other common oils on CCl_4_ induced LF. Healthy male ICR mice were administered with CCl_4_ intraperitoneally at 2.5 ml/kg twice a week for total 3 weeks. Mice were pre-treated with olive oil, soybean oil, corn oil or lard oil. After treatment, histopathological changes were observed using Masson trichrome staining, and alanine aminotransferase (ALT), aspartate aminotransferase (AST), malondialdehyde (MDA), hydroxyproline (HYP) and triglyceride (TG) were measured by commercial kits. The expression of LF related genes was detected by quantitative real-time PCR. We found that soybean oil or olive oil significantly reduced ALT and AST levels in serum, and MDA, HYP and TG levels in the liver, compared with corn oil or lard oil. Moreover, Masson trichrome staining and real-time PCR showed that the mice treated with CCl_4_ dissolved in soybean oil or olive oil had less fibrosis and apoptosis in the liver comparted to the mice treated with CCl_4_ dissolved in corn oil or lard oil. In conclusion, soybean oil but not corn or lard oil exerts protective effects against CCl_4_ induced LF in mice, possibly due to its antioxidant activity.

## Introduction

The liver is important to the homeostasis in human body [1]. However, alcohol, medicine and carbon tetrachloride (CCl_4_) may cause hepatotoxicity and promote liver fibrosis (LF) through the activation of hepatic stellate cells (HSCs) and extracellular matrix (ECM) [2]. LF could further develop to cirrhosis and liver cancer, which eventually cause death [3]. Currently, LF has become a main threat to global human health due to the lack of effective treatment. Therefore, there is an urgent need to control LF to improve patients’ quality of life.

Reactive oxygen species (ROS) released by injured HSCs play an important role in oxidative damage during chronic liver injury and LF [4]. Matrix metalloproteinases (MMPs) and tissue inhibitors of metalloproteinases (TIMPs) are essential to the development of LF [5]. HSCs have been shown to secrete inflammatory cytokines, TGF-β, tumor necrosis factor-α and platelet-derived growth factor (PDGF), leading to the up-regulation of α-smooth muscle actin (α-SMA) as a sign of activation [6]. The degradation of ECM component collagen is helpful to the recovery of LF [7]. However, up-regulated Timp1 and Timp2 expression may contribute to ECM deposition in the liver through inhibiting the activity of MMPs [8]. Moreover, MMP-9 could promote HSCs apoptosis and accelerate the recovery of LF [9]. In addition, with the progression of fibrosis, the expression of PDGF and its receptor PDGFR in the liver is increased [10].

Olive oil, soybean oil and corn oil contain a large amount of unsaturated fatty acids, while lard oil is enriched in saturated fatty acids. Several studies have suggested that saturated fatty acids are more cytotoxic than unsaturated fatty acids *in vivo* [11]. Olive oil, a widely used ω-9 enriched dietary lipid, exhibits hepatoprotective effects on CCl_4_-induced LF [12]. Soybean oil has antioxidative activity and could reduce the risk of many diseases [13]. However, the effects of soybean oil on LF are largely unknown. Therefore, the present study aimed to evaluate the effects of soybean oil and other common oils on CCl_4_ induced LF.

## Methods

### Animals

All animal care and experimental procedures were performed at Hainan Medical University and approved by Animal Care and Use Committee of Hainan Medical University. Healthy male ICR mice (20–22 g) were purchased from the experimental Animal Center of Harbin Medical University and maintained at 22 ± 2°C with the humility of 45 ± 10% and regular 12:12 h light–dark cycle. All animals were given water and food *ad libitum*. A total of 108 mice were allowed to acclimatize for 3 days and then randomly divided into eight groups: (1) olive oil control group (olive oil CCl_4_^−^); (2) soybean oil control group (soybean oil CCl_4_^−^); (3) corn oil control group (corn oil CCl_4_^−^); (4) lard oil control group (lard oil CCl_4_^−^); (5) olive oil CCl_4_ group (olive oil CCl_4_^+^); (6) soybean oil CCl_4_ group (soybean oil CCl_4_^+^); (7) corn oil CCl_4_ group (corn oil CCl_4_^+^); (8) lard oil CCl_4_ group (lard oil CCl_4_^+^). Mice from olive oil CCl_4_^+^, soybean oil CCl_4_^+^, corn oil CCl_4_^+^ and lard oil CCl_4_^+^ groups were injected intraperitoneally (i.p.) with 20% CCl_4_ dissolved in olive oil (AGRIC, Greece), soybean oil (Jiusan Oils & Grains Industries Group Co., Ltd, Heilongjiang, China), corn oil (Jiusan Oils & Grains Industries Group Co., Ltd, Heilongjiang, China) and lard oil (Pork, Heilongjiang, China), respectively. Mice from olive oil CCl_4_^−^, soybean oil CCl_4_^−^, corn oil CCl_4_^−^ and lard oil CCl_4_^−^ groups were injected i.p. with olive oil, soybean oil, corn oil and lard oil, respectively. All mice received a dose of 2.5 ml CCl_4_ solution or oil per kilogram of body weight twice a week for 3 weeks. The mice were euthanized, and their blood and livers were collected immediately and stored.

### Measurement of serum ALT and AST

The blood collected from right heart ventricle was stored at room temperature for 4 h and centrifuged at 3000 rmp at 4°C for 15 min. Serum was separated, and ALT and AST were detected using ELISA kits (JCBIO, Nanjing, China) following the manufacturer’s protocols.

### Histological analysis

Part of the liver tissues were fixed in 4% paraformaldehyde for 48 h at room temperature, paraffin-embedded and sectioned for staining with Masson’s trichrome. The morphology of liver tissues was observed under microscope and the images were analzyed using Image Pro Plus 5.1 (Media Cybernetics, Bethesda, MD, U.S.A.).

### Measurement of hydroxyproline, Malondialdehyde and triglyceride

Part of the liver tissues were washed with cold sterile phosphate-buffered saline (PBS), dried and weighted precisely, and hydroxyproline (HYP), malondialdehyde (MDA) and triglyceride (TG) were measured using assay kits (JCBIO, Nanjing, China) following the manufacturer’s protocols [14].

### Real-time PCR

Total RNA was isolated from the liver tissues using Trizol reagent (Invitrogen, Carlsbad, CA, U.S.A.), followed by reverse transcription to cDNA using a PrimeScriptTM RT kit (TOYOBO, Osaka, Japan). Real-time PCR was performed using primers (synthesized by Sangon, Shanghai, China) listed in Table 1 and SYBR^®^ Premix kit (TOYOBO, Osaka, Japan) according to the manufacturer’s instructions. PCR conditions were: 95°C for 30 s, then 40 cycles of 95°C for 5 s, 60°C for 30 s The data were presented as the fold change in mRNA expression levels and normalized to reference gene GAPDH.

**Table 1:** Primer sequences

Target gene			Primer sequence	
TGF-β1	F	5′	GCCTGAGTGGCTGTCTTTTGA	3′
	R	5′	GCTGAATCGAAAGCCCTGTATT	3′
PDGF	F	5′	GGTCCCATGCCATTAACCAT	3′
	R	5′	CCGTCCTGGTCTTGCAAACT	3′
PDGF-receptor	F	5′	CTTTGTGCCAGATCCCACCA	3′
	R	5′	TCACTCGGCACGGAATTGTC	3′
MMP-2	F	5′	CCCTCAAGAAGATGCAGAAGTTC	3′
	R	5′	TCTTGGCTTCCGCATGGT	3′
MMP-13	F	5′	GTTCAAGGAATTCAGTTTCTTTATGGT	3′
	R	5′	GGTAATGGCATCAAGGGATAGG	3′
TIMP-1	F	5′	GCATGGACATTTATTCTCCACTGT	3′
	R	5′	TCTCTAGGAGCCCCGATCTG	3′
TIMP-2	F	5′	TTCCGGGAATGACATCTATGG	3′
	R	5′	GGGCCGTGTAGATAAACTCGAT	3′
col1α1	F	5′	CCTGGCAAAGACGGACTCAAC	3′
	R	5′	GCTGAAGTCATAACCGCCACTG	3′
α-SMA	F	5′	TCCCTGGAGAAGAGCTACGAACT	3′
	R	5′	AAGCGTTCGTTTCCAATGGT	3′
FasL	F	5′	AAGAAGGACCACAACACAA	3′
	R	5′	TAATCCCATTCCAACCAGAG	3′
TNF-α	F	5′	GTGGAACTGGCAGAAGAG	3′
	R	5′	AATGAGAAGAGGCTGAGAC	3′
GAPDH	F	5′	TGCACCACCAACTGCTTAG	3′
	R	5′	GGATGCAGGGATGATGTTC	3′

### Statistical analysis

All data are presented as the mean ± S.E.M. and analyzed by GraphPad Prism 7 (GraphPad Software, CA, U.S.A.). The significant differences among groups were determined by a one-way ANOVA followed by Dunnett’s *post hoc* test. The *P*<0.05 was considered significant difference.

## Results

### Different effects of olive oil, soybean oil, corn oil and lard oil on serum ALT and AST

**Figure 1:**
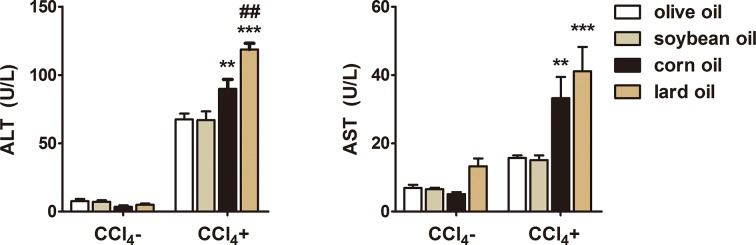
Different effects of olive oil, soybean oil, corn oil and lard oil on serum ALT and AST in CCl_4_-induced liver injury The contents of serum ALT and AST of CCl_4_^−^ and CCl_4_^+^ groups were detected at the end of the 3rd week. The data are shown as mean ± S.E.M. (*n*=9). ^***^P<0.001, ^**^P<0.01, vs olive oil CCl_4_^+^ & soybean oil CCl_4_^+^; ^##^*P*<0.01, vs corn oil CCl_4_^+^.

As most commonly used biochemical markers, ALT and AST reflect liver injury during the progression of LF. Therefore, ALT and AST were detected in serum of control and experimental mice. As shown in Figure 1, mice treated with CCl_4_ had increased levels of ALT and AST compared with control group, indicating that CCl_4_ successfully induced liver injury. However, administration with olive oil or soybean oil significantly decreased ALT and AST in CCl_4_ groups compared with corn oil or lard oil CCl_4_ group. There was no significant difference between olive oil and soybean oil CCl_4_ groups.

### Different effects of olive oil, soybean oil, corn oil and lard oil on liver pathology

**Figure 2:**
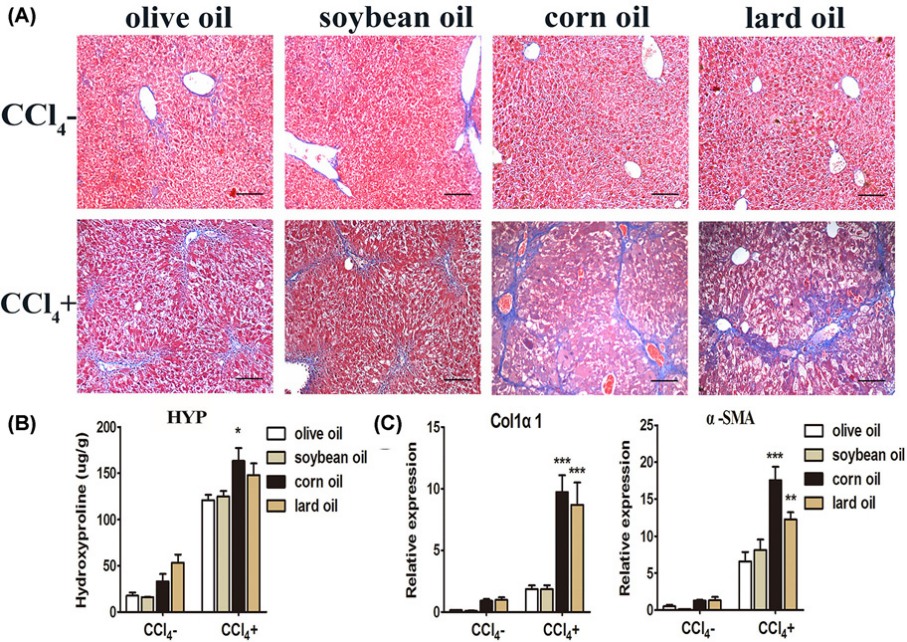
Different effects of olive oil, soybean oil, corn oil and lard oil on CCl_4_-induced pathological changes (**A**) Histopathological analysis of liver sections by Masson’s trichrome staining. The nuclei were stained in dark brown, the cytoplasm were stained in red, and collagen fibres were stained in blue. Scale bars: 100 μm. (**B**) The contents of HYP in each group (**C**) mRNA levels of Col1α1 and α-SMA in each group. The data are shown as mean ± S.E.M. (*n*=9). ^***^P<0.001, ^**^P<0.01, ^*^P<0.05, vs olive oil CCl_4_^+^ & soybean oil CCl_4_^+^.

To observe pathological changes in the livers, we used Masson’s trichrome staining. As shown in Figure 2A, all the livers from CCl_4_^−^ groups showed normal structure with little fibre portal expansion, whereas the livers in CCl_4_^+^ groups exhibited massive fatty changes and pericentral fibrosis with extensive blue-stained fiber. The fatty changes and fiber formation in olive oil or soybean oil CCl_4_ group were similar but much weaker compared with corn oil or lard oil CCl_4_ group. Moreover, the contents of HYP, a specific amino acid for collagen, decreased in olive oil or soybean oil CCl_4_ group compared with corn oil or lard oil CCl_4_ group (Figure 2B). Consistent with these changes, olive oil or soybean oil led to reduced mRNA expression of Col1α1 and α-SMA (Figure 2C).

### Differential effects of olive oil, soybean oil, corn oil and lard oil on the expression of TNF-α and FasL

**Figure 3:**
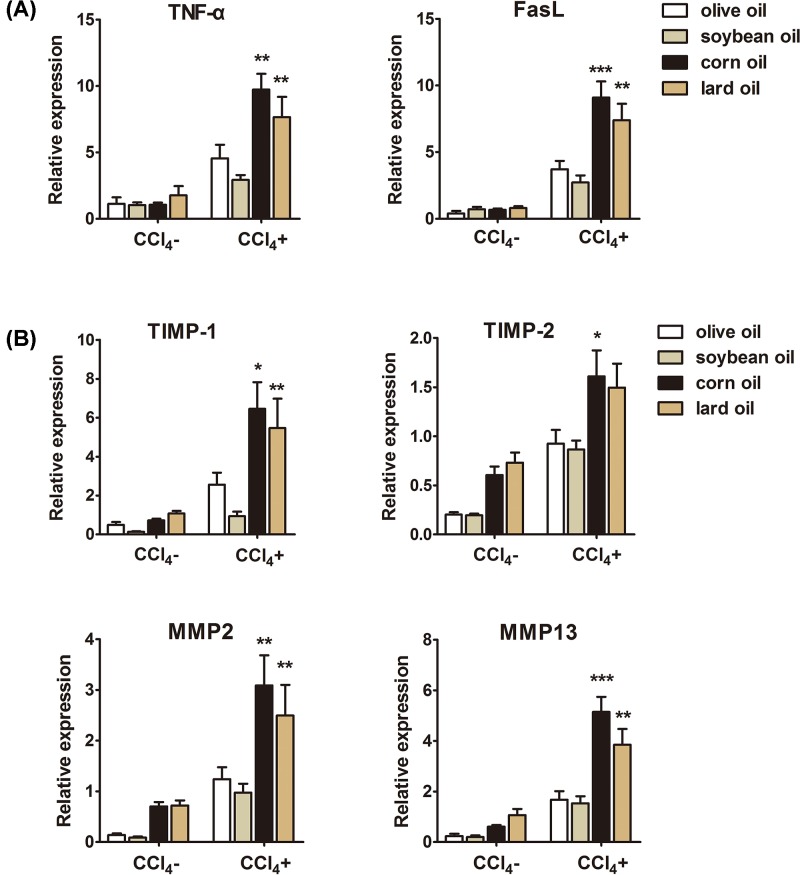
Different effects of olive oil, soybean oil, corn oil and lard oil on apoptosis-related proteins and ECM regulators (**A**) TNF-α and FasL expression decreased in livers from olive oil or soybean oil with CCl_4_-treated mice compared with corn oil or lard oil with CCl_4_-treated ones. (**B**) The expression of TIMP-1, TIMP-2, MMP-2 and MMP-13 decreased in livers from olive oil or soybean oil with CCl_4_-treated mice compared with corn oil or lard oil with CCl_4_-treated ones. The data are shown as mean ± S.E.M. (*n*=9). ^***^P<0.001, ^**^P<0.01, ^*^P<0.05, vs olive oil CCl_4_^+^ & soybean oil CCl_4_^+^.

TNF-α and FasL regulate HSCs apoptosis and aggravate LF [15]. Therefore, we evaluated the effects of four oils on the expression of TNF-α and FasL. As shown in Figure 3A, olive oil or soybean oil significantly decreased the levels of TNF-α and FasL compared with corn oil or lard oil in CCl_4_ treated mice, suggesting that olive oil or soybean oil may reduce HSCs apoptosis to alleviate LF.

### Differential effects of olive oil, soybean oil, corn oil and lard oil on the expression of TIMP and MMP

TIMPs and MMPs play an important role in the degradation of ECM. Therefore, we determined the effects of four oils on the expression of TIMPs and MMPs. As shown in Figure 3B, olive oil or soybean oil with CCl_4_ treatment clearly decreased the expressions of TIMP-1 and TIMP-2, and MMP-2 and MMP-13 compared with those from corn oil or lard oil with CCl_4_ treatment, suggesting that olive oil or soybean oil may alleviate LF by reducing ECM.

### Differential effects of olive oil, soybean oil, corn oil and lard oil on the expression of TGF-β1, PDGF and PDGFR

**Figure 4:**
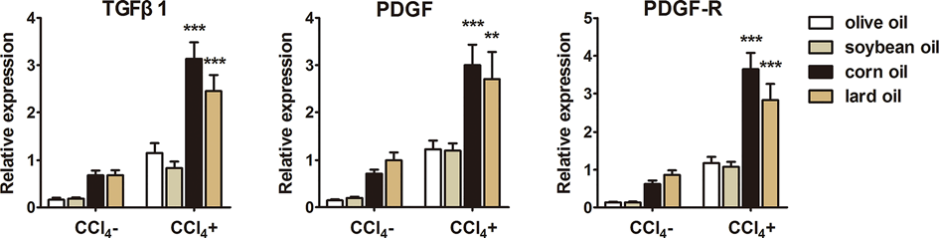
Different effects of olive oil, soybean oil, corn oil and lard oil on mRNA expression of TGF-β1, PDGF and PDGFR Expression of TGF-β1, PDGF and PDGFR decreased in livers from olive oil or soybean oil with CCl_4_-treated mice compared with those from corn oil or lard oil with CCl_4_-treated ones. The data are shown as mean ± SEM (*n*=9). ^***^P<0.001, ^**^P<0.01, vs olive oil CCl_4_^+^ & soybean oil CCl_4_^+^.

TGF-β1, PDGF and PDGFR are related with the proliferation of HSCs. Therefore, we determined the effects of four oils on their expression. Compared with corn oil or lard oil, olive oil or soybean oil significantly reduced the expression of TGF-β1, PDGF and PDGFR in CCl_4_-treated mice (Figure 4), suggesting that olive oil or soybean oil may reduce the proliferation of HSCs to alleviate LF.

### Different effects of olive oil, soybean oil, corn oil and lard oil on hepatic MDA and TG

**Figure 5:**
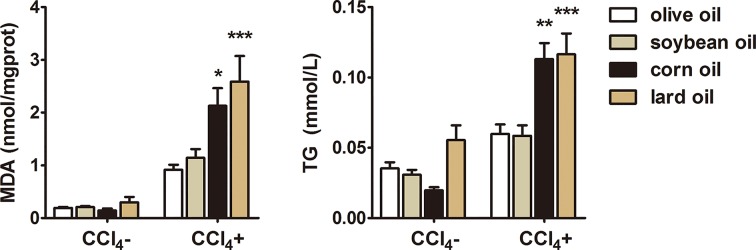
Different effects of olive oil, soybean oil, corn oil and lard oil on hepatic MDA and TG in CCl_4_-induced liver injury The contents of MDA and TG of CCl_4_^−^ and CCl_4_^+^ groups were detected at the end of the 3rd week. The data are shown as mean ± SEM (*n*=9). ^***^P<0.001, ^**^P<0.01, ^*^P<0.05, vs olive oil CCl_4_^+^ & soybean oil CCl_4_^+^.

To detect lipid peroxidation and deposition in the livers, we measured hepatic MDA and TG levels. MDA and TG levels in CCl_4_^+^ groups increased markedly compared with CCl_4_^−^ groups, but decreased significantly in olive oil or soybean oil CCl_4_ group compared with corn oil or lard oil CCl_4_ group (Figure 5). Moreover, there was no significant difference between olive oil and soybean oil CCl_4_ groups.

## Discussion

In the present study, we demonstrated that olive oil and soybean oil had similar effects on CCl_4_-induced LF. Specifically, olive oil and soybean oil significantly alleviated CCl_4_-induced LF as evidenced by reduced serum ALT and AST as hepatic injury indicators; reduced LF pathology accompanied by less HYP in ECM; reduced expression of TNF-α, FasL, α-SMA, TGF-β1, PDGF and PDGFR in the liver; and reduced oxidative stress in the liver with less hepatic MAD and TG.

HYP only exists in collagen and its contents can directly reflect the levels of collagen [16]. In addition, hepatic HYP contents in patients with liver diseases increased proportionally with the progression of LF [17]. Therefore, we considered hepatic HYP as an appropriate indicator of collagen and showed that the contents of hepatic HYP decreased in olive oil or soybean oil CCl_4_ group compared with corn oil or lard oil CCl_4_ group. Furthermore, mRNA expression of Col1α1 and α-SMA reduced in olive oil or soybean oil CCl_4_-treated mice. Our results suggest that the antifibrotic effects of olive oil or soybean oil are partly due to the inhibition of collagen production.

HSCs constantly secrete α1-collagen, TGF-β1, PDGF and PDGFR, which in turn stimulate the activation and proliferation of HSCs to promote LF [18]. Moreover, PDGF, as the strongest mitogen for HSCs, can activate the expressions of MMP-2, -9 and TIMPs through a series of signaling pathways such as PI3K/PKB and p38 MAPK [19]. PDGF could inhibit collagenase activity and ECM degradation [20]. PDGF also promotes HSCs migration to the injured site for the synthesis of ECM [21]. Therefore, we measured the expression of TGF-β1, PDGF and PDGFR in the livers, and found reduced expression of these proteins in olive oil or soybean oil treated mice. These results suggest that the protective effects of olive oil and soybean oil on the liver are probably due to the inhibition of HSCs activation and collagen production.

MMPs and TIMPs play an important role in maintaining the dynamic equilibrium of the production and degradation of ECM, and are involved in LF [22]. We found that TIMPs and MMPs expression markedly reduced in olive oil or soybean oil CCl_4_-treated mice compared with corn oil or lard oil CCl_4_-treated mice. It has been reported that TIMP-1 and -2, and MMP-2 expression significantly increased during LF both in human and in animal models [23]. In addition, TIMP-1 can inhibit the activity of HSCs by inhibiting the apoptosis, which further increases the formation of fibrosis [23]. MMP-13 is involved in liver damage, inflammation, HSC activation and LF [24]. In our study, reduced expression of TIMP-1 and -2, and MMP-2 and -13 in livers treated with olive oil or soybean oil could lead to increased matrix degradation in the liver, consistent with attenuated pathological manifestations and reduced ECM and collagen deposition.

CCl_4_ is known to induce oxidative damage during chronic liver injury and LF [4]. Corn oil could aggravate lipid peroxidation in animals because its composition contains a high proportion of omega-6 PUFAs. Moreover, corn oil could induce oxidative damage in the liver [25]. In our study, olive oil or soybean oil showed significant decrease in MDA levels in CCl_4_-treated mice compared with corn oil or lard oil. Therefore, we could infer that soybean oil, similar as olive oil, might alleviate LF by reducing lipid peroxidation. Furthermore, olive oil or soybean oil led to significant decrease in TG in CCl_4_-treated mice compared with corn oil or lard oil, maybe due to high contents of phenolic compounds and unsaturated fatty acids in soybean oil and olive oil [1]. It has been reported that unsaturated fatty acids could lower serum cholesterol and are recommended as a dietary change to patients [26]. Furthermore, long-term edible olive oil in patients with liver injury could reduce TG [27], consistent with our result of the reduction of TG in olive oil or soybean oil CCl_4_ group. Collectively, these results suggest that olive oil and soybean oil might alleviate LF by reducing lipid deposition compared with corn oil and lard oil, because the total fatty acid composition ratio of the four oils are different. Recent studies showed olive oil could activate Nfr2 pathway to induce cellular antioxidant response and inhibit PERK pathway to prevent endoplasmic reticulum stress, autophagy, and lipogenic response, and essential oils provided hepatoprotection by accelerating acetaminophen harmless metabolism [28–30]. Further studies are needed to reveal novel signaling mechanisms involved in hepatoprotective effects of olive oil and soybean oil.

In conclusion, olive oil and soybean oil show hepatoprotective effects against CCl_4_-induced LF in mice, which is related to the anti-oxidative effects of olive oil and soybean oil. To our knowledge, the present study is the first comprehensive analysis of beneficial effects of olive oil or soybean oil in comparison with corn oil or lard oil on CCl_4_-induced LF. Our findings provide a better understanding of the mechanisms underlying the different effects of dietary lipids on LF.

## Abbreviations

ALT: alanine aminotransferase
α-SMA: α-smooth muscle actin
AST: aspartate aminotransferase
CCl_4_: carbon tetrachloride
ECM: extracellular matrix
HSC: hepatic stellate cell
HYP: hydroxyproline
i.p.: intraperitoneally
LF: liver fibrosis
MDA: malondialdehyde
MMP: matrix metalloproteinase
PDGF: platelet-derived growth factor
TG: triglyceride
TIMP: tissue inhibitors of metalloproteinase
